# One Agency's Contribution to Creating a Culture of Health in a Latinx Community

**DOI:** 10.1089/heq.2018.0056

**Published:** 2019-01-30

**Authors:** Mariela Fernandez, Kimberly J. Shinew

**Affiliations:** ^1^ Department of Parks, Recreation and Tourism Management, College of Behavioral, Social and Health Sciences, Clemson University, Clemson, South Carolina.; ^2^ Department of Recreation, Sport & Tourism, College of Applied Health Sciences, Champaign, Illinois.

**Keywords:** Culture of Health, Hispanic, Latinx, nonprofit, parks

## Abstract

The Robert Wood Johnson Foundations' Culture of Health represents a national call to ensure everyone living in the United States can have a healthy life. This article discusses how organizations outside of the health care sector can participate in creating a Culture of Health by using a nonprofit organization (Little Village Environmental Justice Organization) as an example. The organization's role in policy creation concerning cross-sector partnerships and governance to create equitable communities was of primary importance to this article. The example presented has important public health implications, particularly as it relates to promoting healthy outcomes among urban Latinx community members.

## One Agency's Contribution to Creating a Culture of Health in a Latinx Community

Minority health disparities continue to be a challenge despite community initiatives and reforms in the U.S. health care delivery system.^1–4^ These disparities have been attributed to factors such as racism, limited access to community infrastructure and social services, inadequate healthy food options in neighborhoods, polluted landscapes, and limited political clout with few officials advocating for improved services.^4–9^ If these issues are not addressed, minorities may face considerably shortened life spans.^10,11^

The gravity of the situation has propelled a movement among researchers, the private and public sectors, and community members to seek solutions. Recently, the Robert Wood Johnson Foundation unveiled the Culture of Health Action Framework to address health disparities.^2^ The framework advocates for a society where all individuals can live healthy lives. The framework consists of four action areas: (1) making health a shared value, (2) fostering cross-sector collaborations to improve well-being, (3) creating healthier more equitable communities, and (4) strengthening integration of health services and systems. Embedded in all four action areas is the need for policy creation to foster cross-sector collaborations and ensure more equitable communities. The opportunity to examine how policy creation within the Culture of Health Action Framework can move from principles to practice presented itself in the case of the Little Village Environmental Justice Organization (LVEJO), which operates in Chicago's South Lawndale neighborhood (known as Little Village).

## Little Village

Latest figures indicated that slightly <80,000 residents lived in Little Village, with the majority (83%) identifying as Latinx of Mexican descent.^12^ Residents were predominantly working class and young.^13^ Little Village has historically struggled with issues such as overcrowding in housing and schools,^13^ limited garbage and street services,^14^ and limited green space. Pollution has also been regarded as a problem with the neighborhood having the “2nd worst air quality in the 8 county region of Chicago…Children in this area have the 9th highest rate of lead poisoning of Chicago's 77 community areas with asthma rates of 17%.”^15^

## LVEJO's Role in the Community

LVEJO is a nonprofit organization that uses a social and environmental justice approach to tackle environmental injustices in Little Village. One of LVEJO's major victories involved working alongside community residents and other local nonprofit, public, and private organizations to urge the public and private sectors to remediate a superfund site (land that was contaminated “and identified by the EPA as a candidate for cleanup because it posed a risk to human health and/or the environment”).^16^ The site was associated with respiratory illness, lead in the bloodstream, and skin rashes. Through LVEJO and the community's efforts, the site was transformed into a public park. The strategies used to acquire the park (e.g., educational campaigns, mobilizing residents, confrontational tactics, and democratic leadership) were also used in LVEJO's other projects (e.g., brownfield redevelopment and community gardening).^17^

## Policy Creation to Foster Cross-Sector Collaborations

According to the Culture of Health Action Framework, organizations that foster cross-sector collaborations will institute policies that support such partnerships. LVEJO utilized a holistic approach to collaboration, and partnerships were built with the various stakeholders at the local, state, and national level. In the development of the park, LVEJO partnered with Little Village residents, private businesses owned by Latinxs, health departments to educate residents about the negative health outcomes associated with superfund sites, churches that regarded clean landscapes as healing spaces, and other nonprofit organizations that focused on environmental injustices. LVEJO further partnered with a state senator and the national green organizations (e.g., Sierra Club and Greenpeace). Partnerships with organizations composed of U.S. citizens was vital to protect low-income and undocumented residents as stated by a former LVEJO employee:

In creation of the [a partnership with big green groups], we were able to leverage a lot of resources that organizations like ours don't have. Greenpeace scaled the smokestack in Pilsen and camped there for two days, which is amazing because our community members are like, “I can't afford to get arrested, I have no papers.”^18^

Greenpeace activists were arrested while protecting Latinx residents who could not afford to be arrested.

Tensions often plagued partnerships that did not allow for Latinx voices to be represented. During a 2006 state agreement, which was crafted by policy analysts and lawyers, it appeared that the national green organizations received more voice in the process as Latinx residents and/or organizations were not present. This gave the impression that the national green organizations “were speaking on behalf of other groups or felt empowered enough to speak on behalf of everyone.”^14^ LVEJO's approach was to always allow for residents to speak for themselves.

## Policy and Governance to Create Equitable Communities

To improve equity in neighborhood resources, LVEJO engaged in shaping and ensuring compliance with public policy at the local, state, and federal levels. An example was LVEJO's involvement in shaping the soil and rubble reuse ordinance, where they raised caution over the transfer of dirt from one location to another:

Currently it's [soil and rubble reuse] illegal because more than a century of heavy industrial activity has left the top layer of soil here contaminated to various degrees with PAHs, lead, and other toxins. Under the city's proposal, the dirt dug up through city road, sewer, school, library, and park construction projects could simply be taken to another construction site where it's needed—as long as tests prove it's no more contaminated than what's already there.^19^

Not only did LVEJO shape laws and policies, but they also shed light on selective enforcement where factories situated in low-income minority neighborhoods often received less punishment and/or were allowed to operate longer than factories in middle-income White neighborhoods. At the end of this study, LVEJO also hired a policy director who oversaw environmental policy at the local, state, and federal level.

## Conclusion

Although many health initiatives target communities and individuals, the example of LVEJO sheds light on the importance of working to institute policies at the organizational, local, state, and national level to address health disparities. This was particularly important in the case of Little Village's residents who receive limited city resources and have undocumented residents. LVEJO was able to provide some support and protection of this population by leveraging their partnerships. This allowed for low-income and undocumented residents to be a part of advocating for healthier living spaces, while not being targeted by police and/or U.S. Immigration and Customs Enforcement (ICE) officials.

Most importantly, the example of LVEJO highlights the tenuous relationship between communities of color and the government. Some have argued that government organizations are not designed to address the laws and policies that have negatively affected minority residents' health outcomes.^8,9^ In the case of Little Village, the community's challenges with overcrowding in housing and schools, limited garbage services and green space, and polluted landscapes stem from various historical land use policies. Nonprofit organizations such as LVEJO play a vital role in ensuring that residents of color are protected, especially from the resulting negative health outcomes. As one scholar stated, “When citizens come together to confront a crisis, their collective efforts may influence institutions and processes in which they had no prior leverage.”^20^ LVEJO's role in shaping local, state, and national policy also helped to ensure that Latinx residents had legal recourse to address the health challenges in the community.

It is important to note that not all residents supported LVEJO's work. Some residents preferred development that could create jobs, and development, such as the park, was not perceived as a source of jobs. In addition, LVEJO's social and environmental justice approach was perceived as too radical by some local residents, especially LVEJO's work that challenged local, state, and/or national policies. Despite some residents' concerns with LVEJO's approach, LVEJO's work with other organizations serves as an example of cross-sector partnership that falls outside of the health care sector, but clearly contributes to residents' health outcomes.

The disproportionate negative health outcomes affecting people of color are a serious societal concern. LVEJO's example demonstrated how a nonprofit organization could shape a Culture of Health. Nontraditional approaches are needed given that health disparities continue to affect the public. By enacting policy changes at the organizational, local, state, or national level, other organizations may amplify their success in promoting positive health outcomes.

## Abbreviation Used

LVEJO: Little Village Environmental Justice Organization
